# HDAC10 promotes lung cancer proliferation via AKT phosphorylation

**DOI:** 10.18632/oncotarget.10673

**Published:** 2016-07-18

**Authors:** Yiwei Yang, Yitong Huang, Zhantong Wang, Hsin-tzu Wang, Baoyu Duan, Dan Ye, Chenxin Wang, Ruiqi Jing, Ye Leng, Jiajie Xi, Wen Chen, Guiying Wang, Wenwen Jia, Songcheng Zhu, Jiuhong Kang

**Affiliations:** ^1^ Clinical and Translational Research Center of Shanghai First Maternity & Infant Hospital, Shanghai Key Laboratory of Signaling and Disease Research, Collaborative Innovation Center for Brain Science, School of Life Science and Technology, Tongji University, Shanghai 200092, P. R. China

**Keywords:** lung cancer, histone deacetylase 10 (HDAC10), AKT, cell cycle, cellular localization

## Abstract

Histone deacetylase 10 (HDAC10) is a member of the class II HDACs, and its role in cancer is emerging. In this study, we found that HDAC10 is highly expressed in lung cancer tissues. It resides mainly in the cytoplasm of lung cancer cells but resides in the nucleus of adjacent normal cells. Further examinations revealed that HDAC10 resides in the cytoplasm in multiple lung cancer cell lines, including the A549, H358 and H460 cell lines, but mainly resides in the nucleus of normal lung epithelial 16HBE cells. A leucine-rich motif, R^505^L^506^L^507^C^508^V^509^A^510^L^511^, was identified as its nuclear localization signal (NLS), and a mutant (Mut-505-511) featuring mutations to A at each of its original R and L positions was found to be nuclear-localization defective. Functional analysis revealed that HDAC10 promoted lung cancer cell growth and that its knockdown induced cell cycle arrest and apoptosis. Mechanistic studies showed that HDAC10 knockdown significantly decreased the phosphorylation of AKT at Ser473 and that AKT expression significantly rescued the cell cycle arrest and apoptosis elicited by HDAC10 knockdown. A co-immunoprecipitation assay suggested that HDAC10 interacts with AKT and that inhibition of HDAC10 activity decreases its interaction with and phosphorylation of AKT. Finally, we confirmed that HDAC10 promoted lung cancer proliferation in a mouse model. Our study demonstrated that HDAC10 localizes and functions in the cytoplasm of lung cancer cells, thereby underscoring its potential role in the diagnosis and treatment of lung cancer.

## INTRODUCTION

Lung cancer, 80-85% of which comprises non-small cell lung cancer (NSCLC), is the fastest growing malignant cancer worldwide [1–4]. The molecular mechanism underlying lung cancer development is associated with alterations in signal transduction that result from aberrant expression of oncogenes or tumour suppressor genes [5]. Histone deacetylase (HDAC)-mediated silencing of tumour suppressor genes, such as AKT, GSK3β, c-MYC and p53, has been implicated in cancer development [6–11]. Lung cancer cells expressing high levels of HDAC appear to exhibit greater sensitivity to some HDAC inhibitors than normal cells. An HDAC inhibitor, suberoylanilide hydroxamic acid (SAHA), induced significant growth arrest in NSCLC cells, which may represent a novel approach to the treatment of NSCLC [12, 13]. Studies of the function and molecular mechanisms underlying the involvement of HDACs in lung cancer have provided a clinical basis for targeting HDACs in lung cancer treatment [13].

To date, 18 human HDACs have been identified and have been grouped into classes I-IV. Class I HDACs, including HDAC1, 2, 3, and 8, are ubiquitously expressed and predominantly function as transcriptional repressors in the nucleus. Class II HDACs comprise class IIa (HDAC4, 5, 7 and 9) and class IIb (HDAC6 and 10) HDACs. Class III HDACs are called sirtuins and share little homology with the first two classes. HDAC11 shares some, but not significant, homology with both class I and class II HDACs and is a class IV HDAC. Class IIa HDACs have unique features that enable them to shuttle between the nucleus and the cytoplasm. Unphosphorylated class IIa HDACs remain in the nucleus and repress transcription; however, upon phosphorylation at their amino-terminus, class IIa HDACs shuttle out of the nucleus, which facilitates de-repression of their target genes in the nucleus [14, 15]. HDAC10, which belongs to the class IIb subfamily, has an active deacetylase domain (DAC) and a catalytically inactive leucine-rich domain (LRD) [16–18]. Researchers have begun to elucidate the function of HDAC10 in recent years and determined that it plays a role in homologous recombination [19], promotes autophagy and survival in neuroblastoma cells [20], suppresses cervical cancer metastasis [21] and facilitates the cell cycle [22]. However, whether HDAC10 functions in lung cancer is unknown.

The epidermal growth factor (EGF) receptor is associated with the development of lung cancer, and approximately 14% of NSCLCs harbour mutations in the EGF receptor (EGFR) [23, 24]. EGFR signals mainly through the PI3K/AKT/mTOR kinase cascade, and AKT is the most crucial component of this cascade. AKT is recruited to the plasma membrane and phosphorylated at Thr308 within its catalytic domain by PDK1 (phosphoinositol-dependent kinase 1) before being phosphorylated again at Ser473 by mTORC2 (mammalian target of rapamycin complex 2), which results in full activation of its kinase activity. Multiple phosphatases, including PP2A and PHLPPs, exert direct negative effects on AKT by dephosphorylating Thr308 and Ser473. PTEN exerts an indirect negative effect on AKT by reducing its recruitment to the cell membrane. Aberrant expression and hyperactivation of AKT are associated with lung cancer [25]. However, regulation by phosphorylation does not sufficiently explain how AKT hyperactivation occurs in tumours with normal levels of PI3K/PTEN activity. Mounting evidence indicates that other modifications or protein interactions [25] are involved in AKT activation. Thus far, AKT has been an attractive target in cancer therapy [4, 25, 26].

In the present study, we analysed HDAC10 expression in a lung cancer tissue microarray and found that it was highly expressed in the cytoplasm of lung cancer cells. Further investigation revealed that HDAC10 promotes lung cancer growth, in which AKT is potentially involved.

## RESULTS

### HDAC10 expression and localization in cancer tissue are different from those in adjacent normal tissue

**Figure 1:**
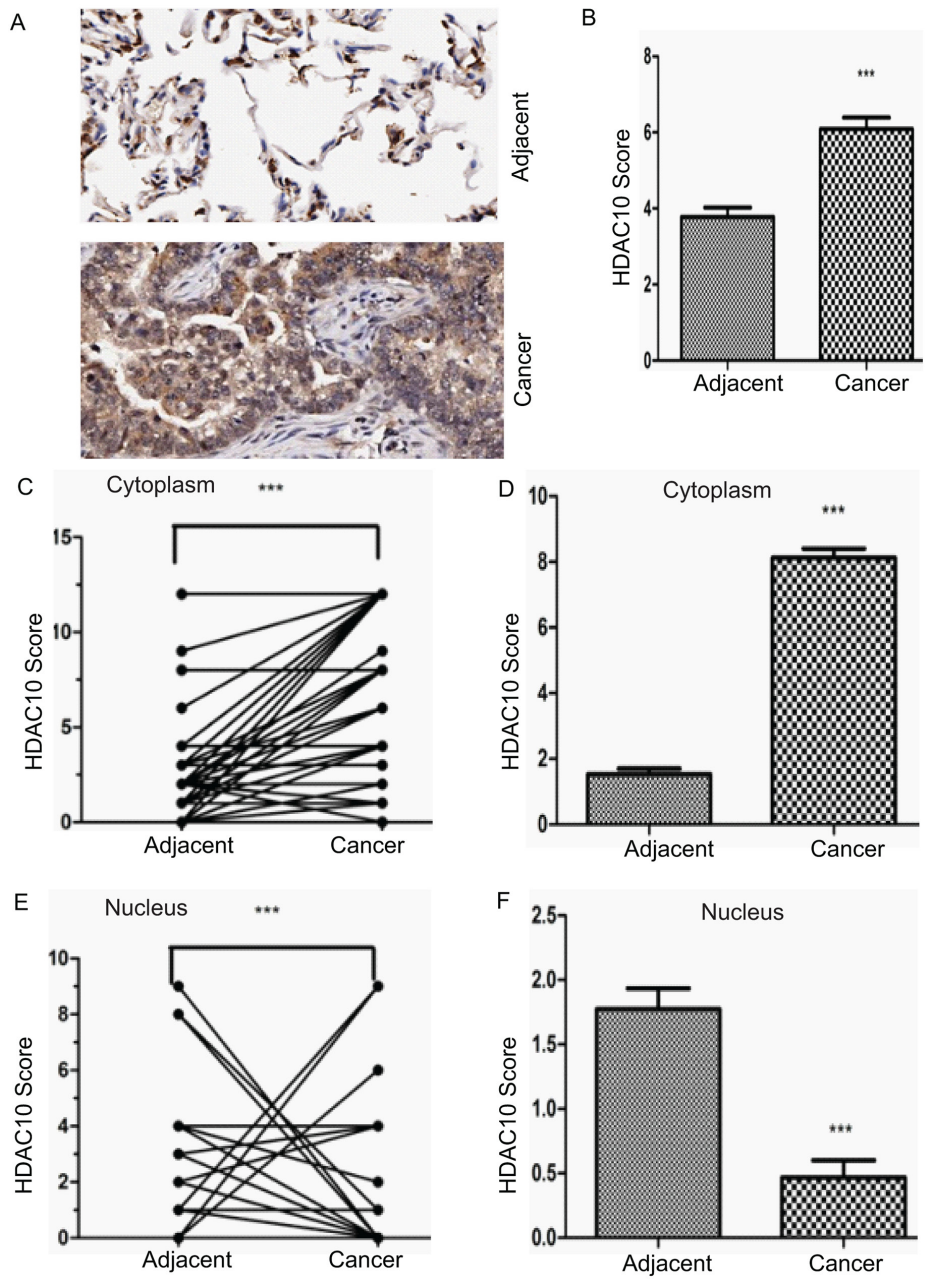
Expression and distribution of HDAC10 in human lung cancer cells **A.** Representative images of HDAC10 expression in lung cancer tissue and corresponding adjacent normal tissue. **B.** Total HDAC10 scores in normal tissue and corresponding tumour tissue. **C.** A plot of scores based on the cytoplasmic expression of HDAC10 in lung cancer tissue and corresponding adjacent normal tissue. The scores of adjacent normal and cancer tissue from the same patient are connected by a line and compared with each other (if the scores of the adjacent tissue of several patients are the same, but the scores of their cancer tissue are different, there will be few lines projecting from one adjacent tissue data point, and vice versa). **D.** Total cytoplasmic HDAC10 scores of adjacent normal and tumour tissue. **E.** A plot of scores based on the nuclear expression of HDAC10 in lung cancer tissue and corresponding normal tissue. **F.** Total nuclear HDAC10 scores of adjacent normal and tumour tissue. ***, p<0.0001. Error bars, standard deviation.

To determine the physiological significance of HDAC10 in human lung cancer, we analysed HDAC10 protein levels in an NSCLC tissue array. The array included cancer tissue samples and corresponding adjacent normal tissue samples from 75 lung cancer patients, including 39 male and 36 female patients ranging from 20 to 84 years of age. 36 patients died during follow-up, and 39 patients survived. Of the 75 patients, 40 had lymph node metastasis, and 35 did not. According to the tumour-node-metastasis (TNM) staging system, 34 of the 75 patients had TNM stage I disease, 12 had stage II disease, 16 had stage III disease and 3 had stage IV disease (data were missing for 10 patients). The levels and localization of HDAC10 in the tissue array specimens were assessed using antibody staining (Figure 1A). Statistical analysis revealed that the levels of HDAC10 in lung cancer tissue were significantly higher than in normal tissue (Figure 1B). We found that the cytoplasmic signal of HDAC10 in cancer tissue was higher than in normal tissue (Figure 1C) and that the difference between the signals was statistically significant (Figure 1D). However, the nuclear signal of HDAC10 in cancer tissue was lower than in normal tissue (Figure 1E), and the difference between the signals was statistically significant (Figure 1F). The analysis yielded very interesting results that cytoplasmic HDAC10 expression in cancer tissue was significantly higher (p=0.0001) than in normal tissue, and nuclear HDAC10 expression in cancer tissue was significantly lower (p=0.0001) than in normal tissue. These data suggest that cytoplasmic HDAC10 expression accounts for HDAC10 overexpression in cancer tissue and that HDAC10 exhibits distinct localization in normal tissue and cancer tissue.

### HDAC10 localization is cell-type dependent

**Figure 2:**
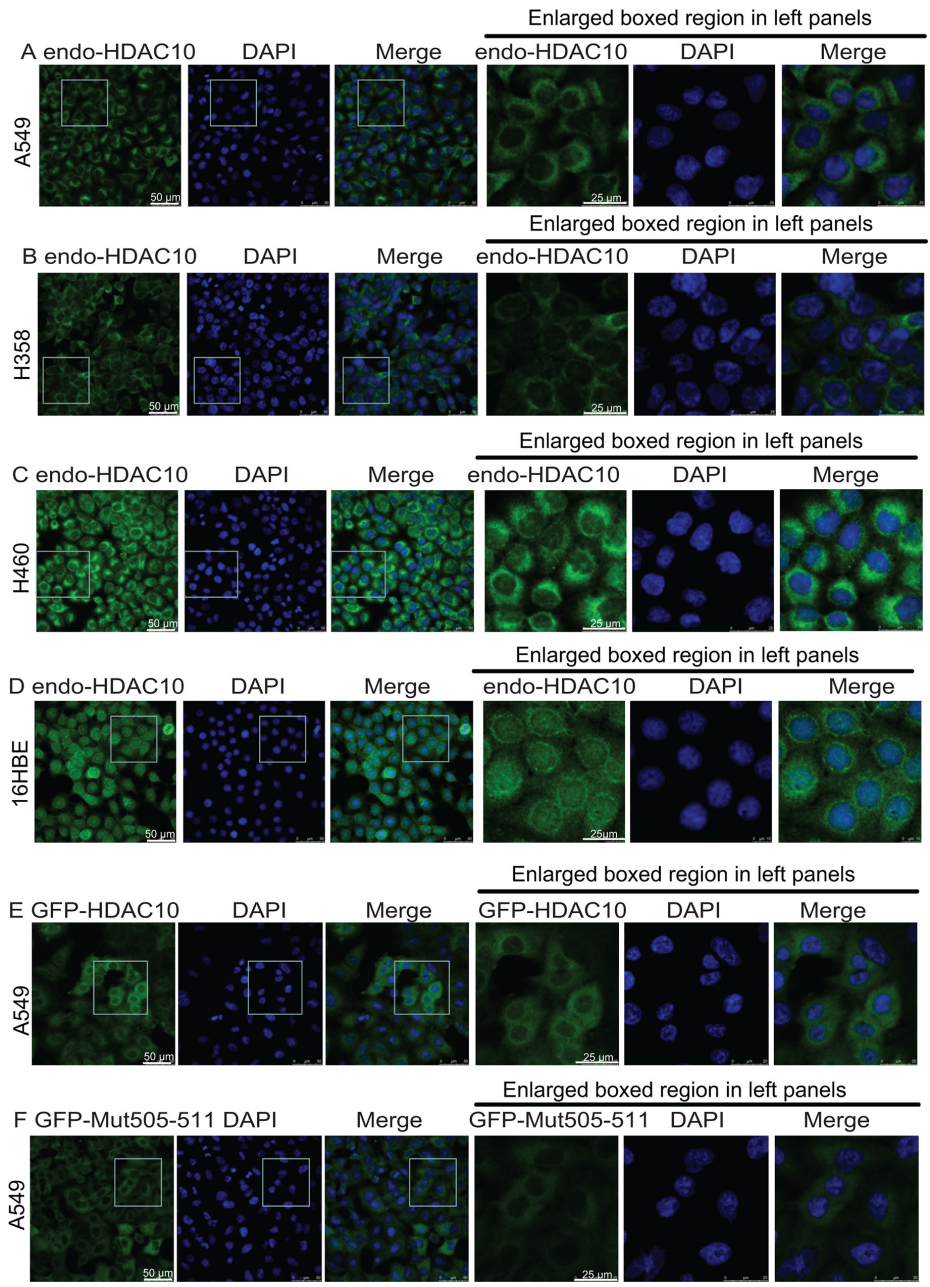

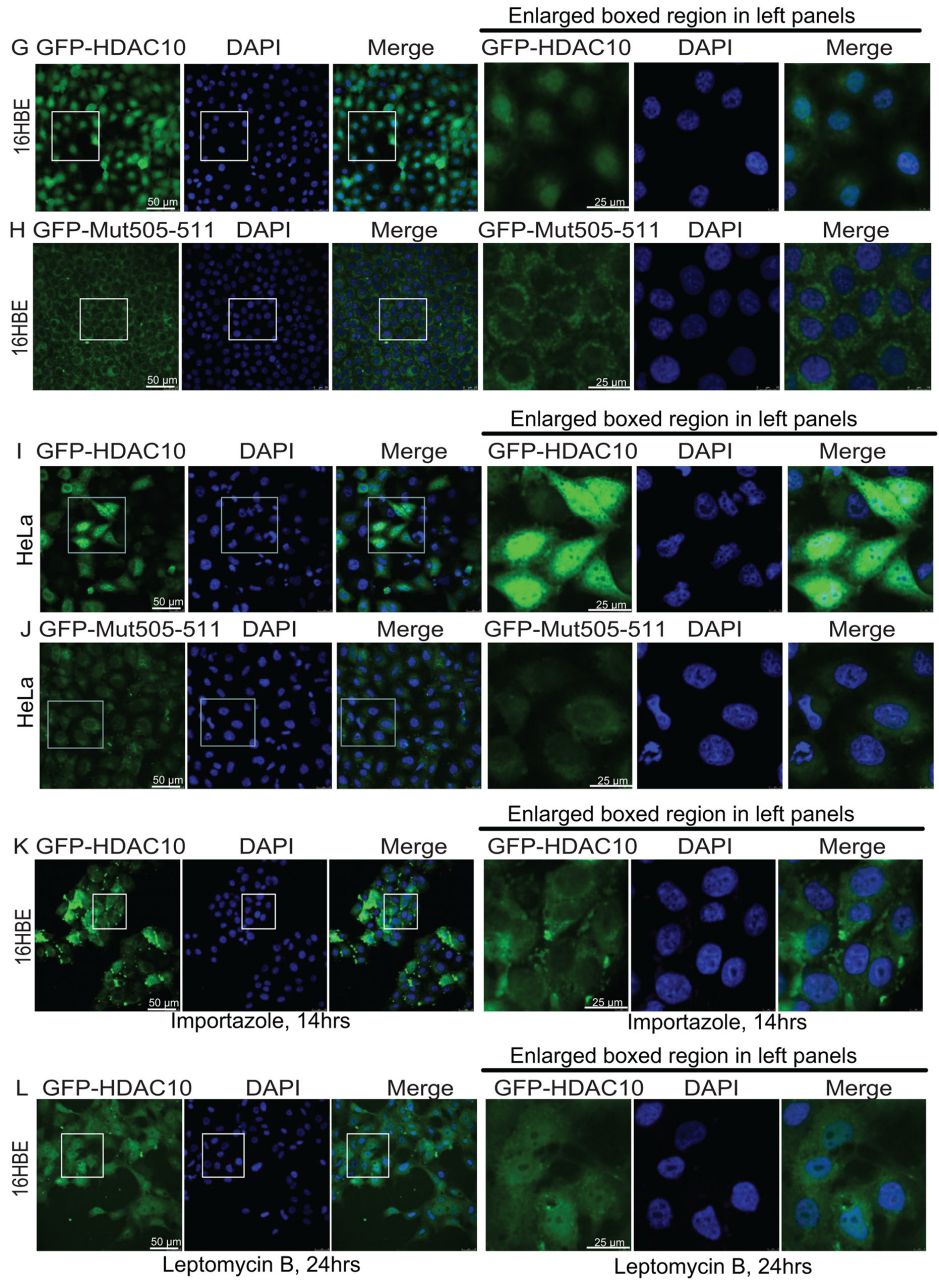
HDAC10 localization in lung cancer cell lines and lung epithelial cell line **A, B, C, D.** Localization of endogenous HDAC10 in the A549 (A), H358 (B), and H460 (C) lung cancer cell lines and the normal lung epithelial cell line, 16HBE (D). **E, F, G, H, I, J.** Localization of wildtype GFP-HDAC10 and GFP-Mut505-511 in the A549 (E and F), 16HBE (G and H) and HeLa (I and J) cell lines. K and L, Localization of GFP-HDAC10 in 16HBE cells upon treatment with Importazole (K) or Leptomycin B (L) for indicated times. Scale bar, as indicated.

HDAC10 resides mainly in the cytoplasm of lung cancer cells but resides in the nucleus of adjacent normal cells. To verify this observation, we examined the distribution of endogenous HDAC10 in multiple lung cancer cell lines, including the A549 (Figure 2A), H385 (Figure 2B) and H460 (Figure 2C) cell lines, and a normal lung cell line, 16HBE (Figure 2D). The results reveal that in all three lung cancer cell lines, HDAC10 resided in the cell cytoplasm (Figure 2A, 2B and 2C). However, in the 16HBE cell line, HDAC10 mainly resided in the nucleus and nuclear membrane. These results suggest that HDAC10 localization is cell-type dependent.

We examined the primary sequence of HDAC10 and found that the following motif in its leucine-rich domain (LRD) is potentially associated with its cellular localization: R^505^L^506^L^507^C^508^V^509^A^510^L^511^. A mutant, in which the original R and Ls within the motif were mutated to As (A^505^A^506^A^507^C^508^V^509^A^510^A^511^), was created and named as Mut505-511. Lentiviral plasmids expressing GFP-fused HDAC10 (GFP-HDAC10) and Mut505-511 HDAC10 (GFP-Mut505-511) were generated. Their expression in A549 cells revealed that GFP-HDAC10 and GFP-Mut505-511 behaved similarly to endogenous HDAC10 and were localized in the cytoplasm (Figure 2E and 2F). In the normal lung epithelial cell line, 16HBE, GFP-HDAC10 was mainly localized in the nucleus (Figure 2G). However, GFP-Mut505-511 was localized to the cytoplasm in 16HBE cells and was therefore nucleus-localization defective (Figure 2H). We subsequently examined GFP-HDAC10 localization in cervical epithelial cancer HeLa cells and found that it was localized in the nucleus in approximately 60% of cells and in the cytoplasm in approximately 40% of cells (Figure 2I). GFP-Mut505-511 was localized in the cytoplasm in all HeLa cells (Figure 2J), further confirmed that GFP-Mut505-511 was nucleus-localization defective.

We found that treatment with Importazole [27], an inhibitor of the transport receptor Importin-β, blocked the nuclear localization of HDAC10 in 16HBE cells (Figure 2K *vs* 2G), while Leptomycin B, an XPO1 inhibitor, had no obvious effect on HDAC10 localization in 16HBE cells (Figure 2L).

The above data suggest that HDAC10 localization is cell-type dependant and cytoplasmic in lung cancer cells and that the R^505^L^506^L^507^C^508^V^509^A^510^L^511^ motif is the NLS of HDAC10.

### Overexpression of HDAC10 promotes lung cancer growth

**Figure 3:**
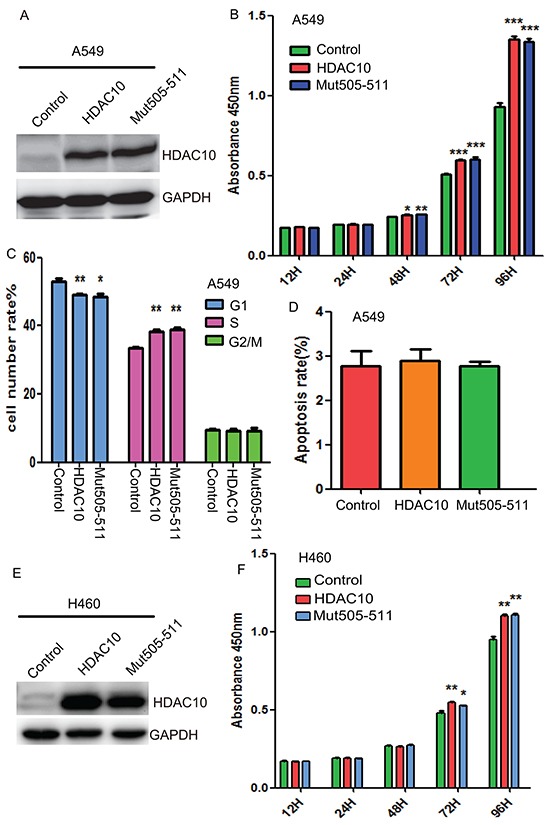
HDAC10 overexpression promotes lung cancer growth **A.** Western blot analysis of HDAC10 and Mut505-511 overexpression. **B.** CCK8 assay measurements of cell growth in A549 cells upon HDAC10 and Mut505-511 overexpression. **C.** Cell cycle analysis with PI staining upon HDAC10 and Mut505-511 overexpression in A549 cells. **D.** Analysis of apoptosis with PI and Annexin V co-staining in A549 cells. **E.** Western blot analysis of HDAC10 and Mut505-511 overexpression in H460 cells. **F.** CCK8 assay measurements of cell growth in H460 cells upon HDAC10 and Mut505-511 overexpression. *, p<0.05; **, p<0.01. ***, p<0.001, Error bars, standard deviation.

Given the elevated expression of HDAC10 in lung cancer tissue, we evaluated the effects of HDAC10 overexpression on cell growth, cell cycle activity and apoptosis in lung cancer cells. Wildtype HDAC10 and the nucleus-localization-defective mutant Mut505-511 were successfully overexpressed in A549 cells (Figure 3A). We found that overexpression of HDAC10 significantly promoted A549 cell growth (Figure 3B). Mut505-511 promoted cell growth in the same manner as the wildtype. Cell cycle analysis suggested that upon overexpression of either wildtype HDAC10 or Mut505-511, the percentage of cells in G1 phase decreased, while the percentage of cells in S phase increased significantly (Figure 3C). In contrast to its effects on cell growth, HDAC10 overexpression did not have any effects on apoptosis (Figure 3D). We found that HDAC10 and Mut505-511 (Figure E) also promoted the growth of lung cancer H460 cells (Figure 3F). These results suggest that HDAC10 promotes lung cancer cell growth and these effects are exerted solely by cytoplasmic HDAC10.

### HDAC10 knockdown induces G1 arrest and apoptosis in lung cancer cells

**Figure 4:**
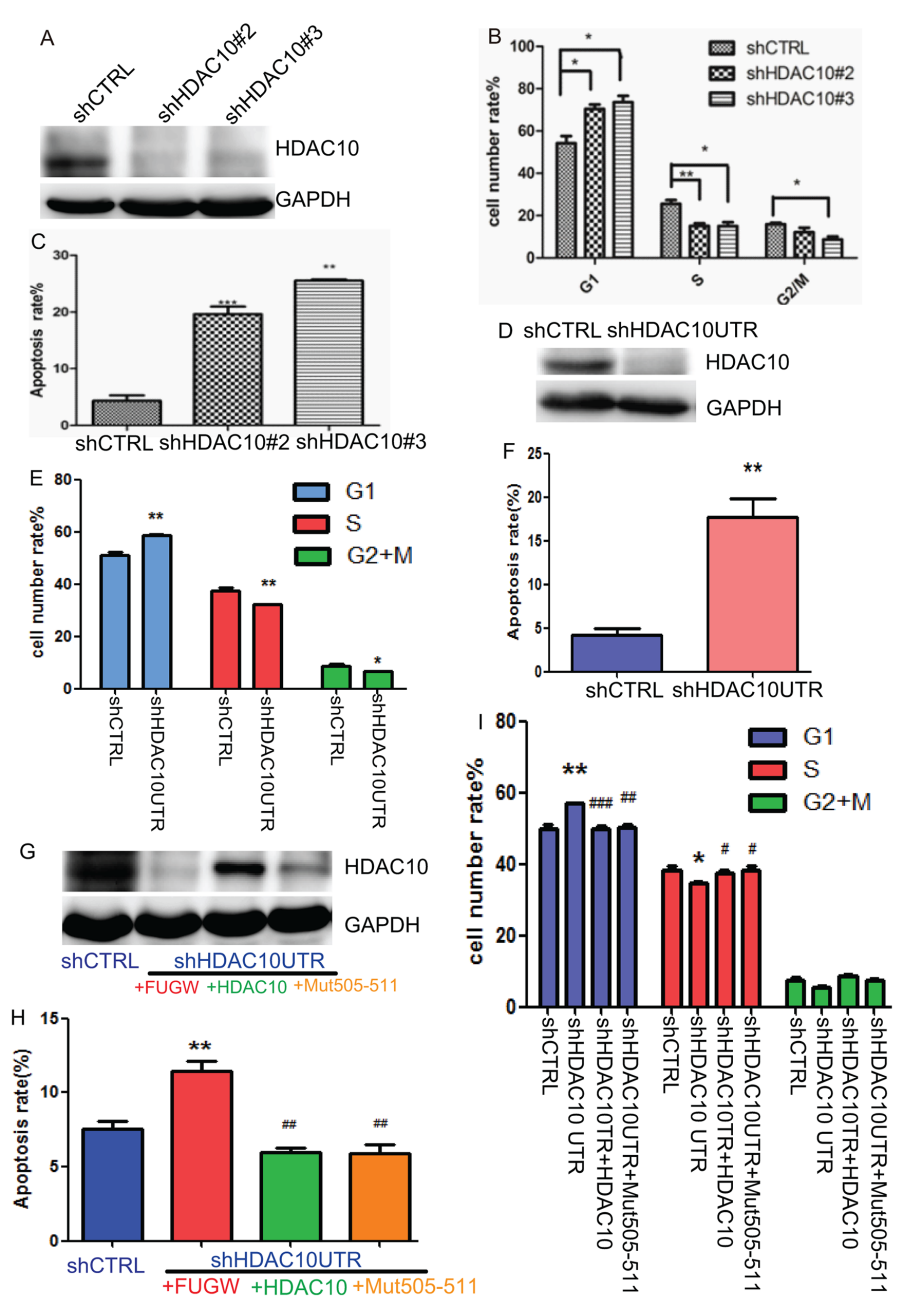
HDAC10 knockdown-induced cell cycle arrest and apoptosis in lung cancer cells **A.** Western blot analysis of HDAC10 knockdown by shRNA targeting HDAC10 coding sequences. **B.** Cell cycle analysis of A549 cells upon HDAC10 knockdown for 72 hours. **C.** Analysis of apoptosis upon HDAC10 knockdown for 96 hours. **D.** Western blot analysis of HDAC10 knockdown by shRNA targeting of the HDAC10 3′ UTR (shHDAC10UTR) region. **E.** Cell cycle analysis of A549 cells upon HDAC10 knockdown by shHDAC10UTR. **F.** Apoptosis analysis of A549 cells upon HDAC10 knockdown by shHDAC10UTR. **G.** Restored expression of HDAC10 and Mut505-511 in A549 cells subjected to HDAC10 knockdown by shHDAC10UTR. **H.** Effects of HDAC10 and Mut505-511 expression on the apoptosis in A549 cells subjected to HDAC10 knockdown by shHDAC10UTR. **I.** Effects of HDAC10 and Mut505-511 expression on cell cycle in A549 cells subjected to HDAC10 knockdown by shHDAC10UTR. *, p<0.05, relative to shCTRL, **, p<0.01. ***, p<0.001. #, p<0.05, relative to shHDAC10UTR; ##, p<0.01, ###, p<0.001. Error bars, standard deviation.

We subsequently studied how depletion of HDAC10 affects the behaviour of lung cancer cells. Two shRNAs that were previously shown to be effective in knocking down HDAC10 [21] were cloned into the lentiviral vector pLKO.1 (shHDAC10 #2 and shHDAC10 #3) to facilitate lentivirus-mediated HDAC10 knockdown in A549 cells (Figure 4A), which resulted in significant G1 arrest (Figure 4B) and apoptosis in these cells (Figure 4C). To determine whether these effects are caused specifically by HDAC10 depletion, we created another shRNA against the 3′UTR region of HDAC10 (shHDAC10UTR). This shRNA targeted only endogenous HDAC10 and facilitated restoration of the ectopic expression of HDAC10 or its mutant. HDAC10 knockdown by this shRNA was efficient (Figure 4D) and resulted in cell cycle arrest (Figure 4E) and apoptosis (Figure 4F). Importantly, restoration of wildtype HDAC10 or Mut505-511 expression (Figure 4G) rescued the cell cycle arrest (Figure 4H) and apoptosis (Figure 4I) induced by HDAC10 knockdown. These data indicate that HDAC10 is required for lung cancer cell growth and survival and that its knockdown induces G1 arrest and apoptosis in lung cancer cells.

### HDAC10 regulates the cell cycle and apoptosis in lung cancer cells via AKT

**Figure 5:**
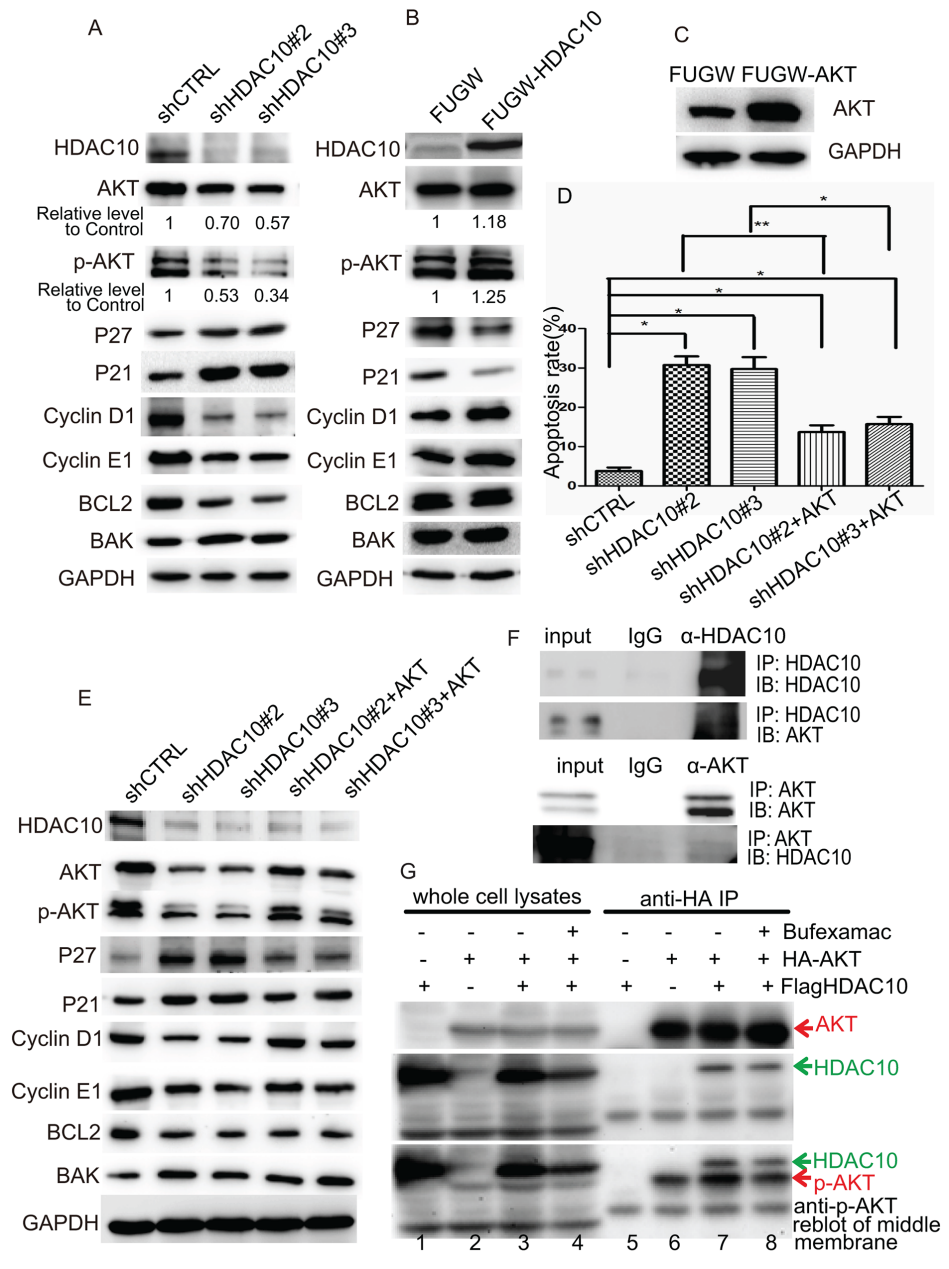
HDAC10 regulates lung cancer growth through AKT **A** and **B.** Western blot analysis of the protein levels of cell cycle regulators, apoptosis regulators and AKT in A549 cells upon HDAC10 knockdown (A) and HDAC10 overexpression (B). **C.** Western blot analysis of the overexpression of FUGW-AKT in A549 cells. **D.** Statistical analysis of the apoptosis levels in A549 cells subjected to HDAC10 knockdown and the rescuing effect by AKT overexpression. *, p<0.05; **, p<0.01. **E.** Western blot analysis of the protein levels of cell cycle regulators, apoptosis regulators and AKT in A549 cells subjected to HDAC10 knockdown and the rescuing effect by AKT overexpression. **F.** Reciprocal co-IP of HDAC10 and AKT in A549 cells. The assay was repeated for three times. **G.** co-IP assay examining the interactions of HDAC10 in 293T cells. Equal amounts of IP products were loaded and subjected to anti-AKT or anti-HDAC10 blotting. The membrane used for anti-HDAC10 blotting (middle panel) was reblotted with anti-p-AKT antibodies (bottom panel). The assay was repeated for three times.

Next, we focused on how HDAC10 regulates lung cancer cell growth and survival. We measured the levels of key cell cycle regulators, including cell cycle inhibitors, such as P21 and P27; cell cycle promoters, such as Cyclin E1 and Cyclin D1; and apoptosis-related proteins, such as the anti-apoptotic BCL2 and pro-apoptotic BAK. Given the central role played by AKT kinase in the EGFR pathway in lung cancer, both total AKT levels and its Ser473-phosphorylated form were also measured. We found that upon HDAC10 knockdown, P27 and P21 were upregulated, and Cyclin D1 and Cyclin E1 were downregulated (Figure 5A). The level of AKT phosphorylation at Ser473, which is essential for its full activation [25], decreased dramatically compared to the decrease in its total level. The level of the pro-apoptotic protein BAK increased, while that of the anti-apoptotic protein BCL2 decreased, which was consistent with the results of the apoptosis assay. Upon overexpression of HDAC10, P27 and P21 were downregulated, and Cyclin D1 and Cyclin E1 were upregulated (Figure 5B). The expression of the abovementioned apoptosis-related factors remained the same. The levels of both phosphorylated AKT and total AKT increased slightly upon HDAC10 overexpression. The changes in the levels of these proteins were consistent with and supported the results of previous functional assays, suggesting that HDAC10 exerts regulatory effects on the levels of AKT, particularly the phosphorylated form of AKT, in lung cancer cells.

We examined whether HDAC10 regulates the cell cycle and apoptosis through AKT. We constructed an AKT-overexpression lentiviral vector (FUGW-AKT) and restored AKT expression in cells in which HDAC10 was knocked down (Figure 5C). Apoptosis analysis revealed that upon restoration of AKT, the apoptosis induced by HDAC10 knockdown was significantly rescued (Figure 5D). The changes in the expression of the cell cycle and apoptosis factors induced by HDAC10 knockdown were also rescued by AKT restoration (Figure 5E). These results suggest that the function of HDAC10 in lung cancer is partially mediated by AKT activation.

Next, we investigated how HDAC10 works with AKT with respect to cell cycle and cell survival regulation. We carried out a reciprocal co-immunoprecipitation assay in A549 cells, the results of which suggest that HDAC10 and AKT interact with each other in A549 cells (Figure 5F). We investigated whether the overexpressed HA-AKT and Flag-HDAC10 in 293T cells interact with each other. HA beads did not pull down Flag-HDAC10 (Figure 5G, lane 5) but did pull down HA-AKT (Figure 5G, lane 6), and Flag-HDAC10 was immunoprecipitated by HA beads upon HA-AKT expression (Figure 5G, lane 7), suggesting that HDAC10 interacts with AKT. More importantly, HDAC10-AKT interactions were reduced upon treatment with the class IIb HDAC inhibitor Bufexamac (Figure 5G, lane 7 vs 8). We also observed that in immunoprecipitated AKT, Ser473 phosphorylation increased upon HDAC10 expression and decreased upon HDAC10 inhibition (Figure 5G, bottom panel, lanes 6, 7 and 8). This observation is consistent with the observations pertaining to A549 cells (Figure 5A and 5B).

### HDAC10 expression promotes tumourigenesis *in vivo*

**Figure 6:**
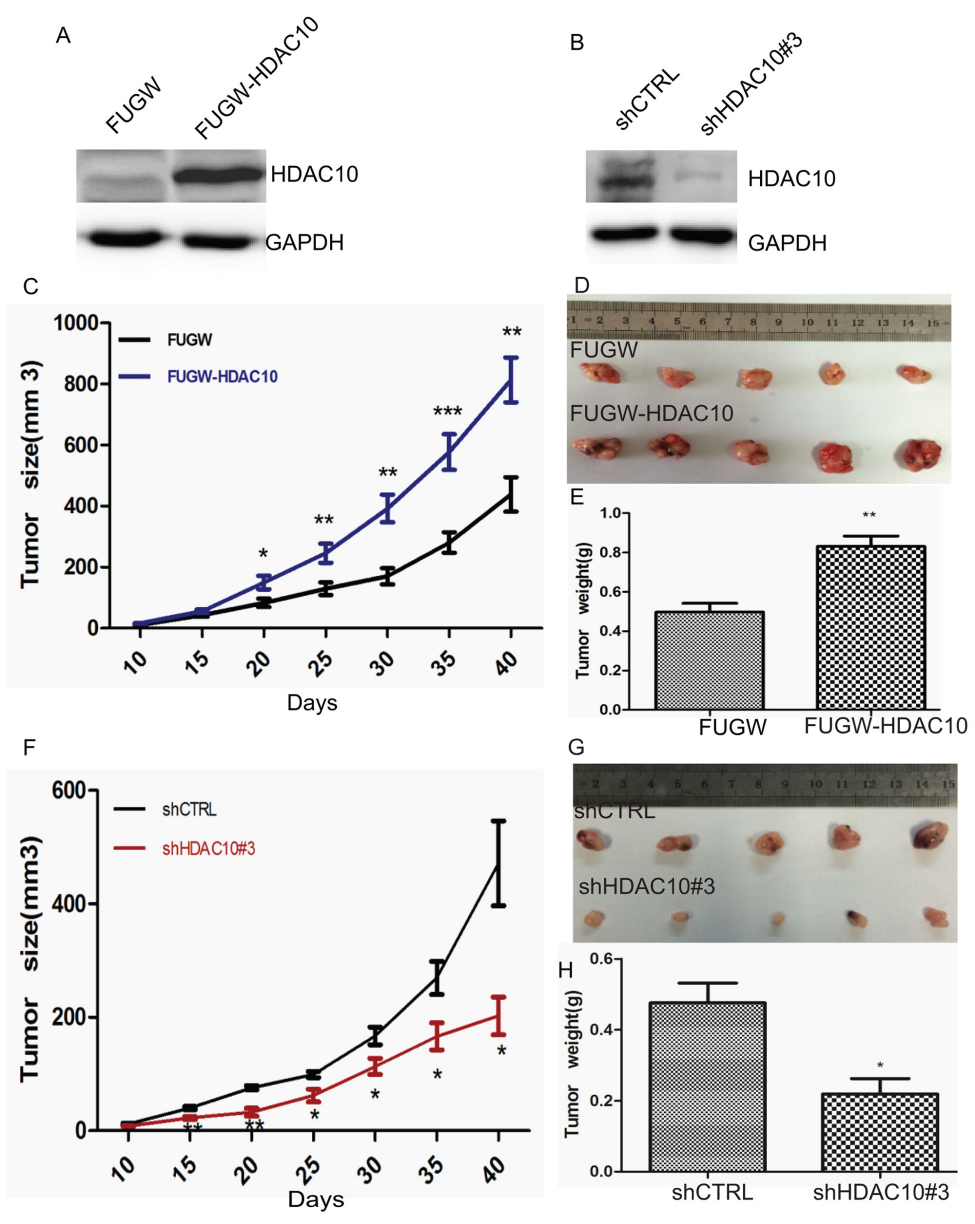
HDAC10 expression affects lung cancer cell growth *in vivo* **A** and **B.** Western blot analysis of HDAC10 overexpression and knockdown in cells prior to transplantation. Tumour growth curve of A549 cells characterized by HDAC10 overexpression (FUGW-HDAC10) **C** and knockdown (shHDAC10 #3) **F.** Up to 5 × 10^6^ and 1 × 10^7^ HDAC10-overexpression and HDAC10-knockdown cells, respectively, were injected per mouse. Similar amounts of control cells (FUGW or shCTRL) were injected at the same time. The tumours were monitored, and their volumes were calculated at 5-day intervals. Representative images of tumours isolated from the mice at 40 days post-injection **D** and **G.**; the weights of the tumours were analysed **E** and **H.** *, p<0.05; **, p<0.01. Error bars, standard deviation.

The tissue microarray data and cell function analysis results suggest that HDAC10 plays an important role in cell growth and survival in lung cancer. Next, we determined whether HDAC10 affects lung cancer growth *in vivo.* HDAC10-overexpression (FUGW-HDAC10) (Figure 6A) and HDAC10-knockdown cells (shHDAC10#3) (Figure 6B) were injected into mice, as were cells with empty vectors. Beginning on the date of injection, tumour growth was observed every day and measured every 5 days. We found that tumours comprising HDAC10-overexpressing cells grew significantly faster than those comprising control cells (Figure 6C). Six days after cell injection, the mice featuring tumours induced by HDAC10 overexpression exhibited successful tumour transplantation, while the control mice did not exhibit successful tumour transplantation until 10 days after cell injection. In the HDAC10-knockdown group, tumour initiation required more cell injections, and these tumours grew significantly more slowly than those initiated by the control cells (Figure 6F). At 40 days after injection, the tumours were isolated and weighed. The HDAC10-overexpressing cells produced significantly larger tumours than the control cells (Figure 6D and 6E), and the HDAC10-knockdown cells produced significantly smaller tumours than the control cells (Figure 6G and 6H). These animal experiments confirmed that HDAC10 promotes and is required for tumour growth *in vivo*, and these results were consistent with previous observations regarding the function of HDAC10 in lung cancer cell lines.

## DISCUSSION

Current chemotherapy regimens for advanced NSCLC elicit only modest increases in survival time, largely because symptoms occur only when the disease has reached an advanced stage, and drug resistance is inherent in tumour cells before therapy is initiated [5, 28]. Considerable effort has been devoted to developing solutions to these problems [3]. Combination therapy for cancer featuring HDAC inhibitors and other targeted inhibitors, such as EGFR inhibitors, may enhance treatment efficacy and improve survival times [3]. In this study, we found that HDAC10 is highly expressed in lung cancer cells and is required for tumour growth and survival. These findings are consistent with those mentioned in a recent report stating that HDAC10 regulates the cell cycle [22]. Another recent study found that HDAC10 promotes cell autophagy, which is a major mechanism associated with cancer cell survival and drug resistance [29]. We previously reported that HDAC10 inhibits the metastasis of cervical cancer [20, 30]. These reports suggest HDAC10 performs a variety of functions in different types of cancer. This study reveals that HDAC10 functions in lung cancer cell growth and survival and highlights the promise HDAC10 inhibition holds for improving survival times and eliminating drug resistance in lung cancer.

With the exception of HDAC8, all class I HDAC members form the catalytic core of several higher-order transcriptional repression complexes in the nucleus, including the Sin3A, NuRD, CoREST, and NODE complexes [31–33]. The functional diversity of class I HDACs is associated with the complexity of these complexes in the nucleus. The diverse functions of class II HDACs are more complicated because class II HDACs shuttle in and out of the nucleus. In the nucleus, these HDACs bind to the promoters of their target genes as components of co-repressor complexes [21]. Their translocation from the nucleus to the cytoplasm results not only in elimination of their original transcriptional repression in the nucleus but also in deacetylation of non-histone proteins in the cytoplasm, both may exert pleiotropic effects on cell behaviour [16, 18]. HDAC10 localization in cancer is unknown, and we report for the first time that HDAC10 is located in the cytoplasm in lung cancer cells. This is important with respect to understanding the mechanism by which HDAC10 functions in this type of cancer. With extensive screening, we identified a small region that is essential for the nuclear distribution of HDAC10. This enable us and other groups to further elucidate the cytoplasmic and nuclear roles of HDAC10 in future studies. Protein analysis programs suggest that another motif, L^556^SCILGLVLP^565^, may be a nuclear-exportation signal; however, our experimental data (data not shown) suggest that this motif is not associated with localization regulation. The function of the LRD of HDAC10 has never been reported. Our study showed for the first time that the LRD contains an intrinsic signal that drives HDAC10 into the nucleus. We have sequenced HDAC10 coding region in the A549, H358 and H460 cancer cell lines and found that its NLS was not mutated in these cell lines, which raises the question of how HDAC10 localization is modified in lung cancer. Our preliminary data (data not shown) suggest that the N-terminus of HDAC10 drives the protein from the nucleus to the cytoplasm because deletion of the first 200 residues of its N-terminus changes its localization from the cytoplasm to the nucleus. Based on our results and those of other reports regarding the localization of HDAC10, we hypothesize that the cellular localization of HDAC10 is determined by interplay between its N-terminus and NLS.

We found that HDAC10 knockdown decreases AKT phosphorylation and that overexpression of AKT rescues the cell cycle arrest and apoptosis elicited by HDAC10 knockdown, suggesting that AKT is associated with the function of HDAC10 in lung cancer. We found that HDAC10 interacts with AKT. The interaction between HDAC10 and AKT was decreased upon HDAC10 inhibition. HDAC10 overexpression promoted phosphorylation of AKT, and HDAC10 inhibition suppressed phosphorylation of AKT. The histone deacetylase Sirt1 has been reported to deacetylate AKT [7, 34, 35]. The mechanism by which HDAC10 regulates AKT phosphorylation remains unknown. Perhaps HDAC10 deacetylases AKT, thereby promoting AKT phosphorylation. We have not obtained data to support this hypothesis yet, but we cannot exclude the possibility.

In summary, we observed the increased expression and distinct distribution of HDAC10 in lung cancer cells and identified an intrinsic and critical sequence that is associated with HDAC10 nuclear localization. We demonstrated that HDAC10 promotes lung cancer cell growth and survival and AKT is potentially involved in the process. This study suggests that HDAC10 expression and localization may serve as valuable prognostic markers or potential therapeutic targets in patients with lung cancer.

## MATERIALS AND METHODS

### Tissue microarrays and immunostaining evaluation

Lung cancer tissue microarrays were purchased from the National Engineering Center for BioChips in Shanghai, China. The expression of HDAC10 in these tissues was evaluated via immunohistochemical staining with an HDAC10-specific antibody. Staining was scored according to staining intensity and the percentage of cells stained. Final staining scores were calculated as the products of staining intensity multiplied by the percentages of stained cells. For immunostaining, cells were grown on coverslips and washed three times with Dulbecco's phosphate-buffered saline (D-PBS) before undergoing fixation for 10 min in D-PBS plus 1% paraformaldehyde at room temperature. After fixation, the cells were blocked with 5% BSA in PBS/Tween 20 for 45 min, probed overnight with primary antibodies diluted in 5% BSA, and then probed with dye-conjugated secondary antibodies after three washes. The coverslips were stained with DAPI and mounted for microscopic examination.

### Cell culture and chemical treatment

The human lung cancer cell lines A549, H358, and H460 and the lung epithelial cell line 16HBE were purchased from the Cell Bank at the Shanghai Institutes for Biological Sciences of the Chinese Academy of Sciences and were cultured in 1640 medium supplemented with 10% foetal bovine serum and 1% double antibiotics, as instructed by their provider. HEK293T and HeLa cells were cultured in DMEM and 10% foetal bovine serum (ExCell Bio, Shanghai, China) supplemented with 1% double antibiotics. All cells were maintained at 37°C with 5% CO2. The cells were treated with Importazole (ab146155) [27], Leptomycin B (ethanol solution, ab120501) or Bufexamac (S3023, Selleck, Shanghai, China) for the indicated times.

### Plasmids, transfections, and virus production

A plasmid to facilitate overexpression of HDAC10 was constructed by cloning the HDAC10 coding sequence into an FUGW lentiviral vector. The lentiviral vector pLKO.1 was used for gene knockdown. The two target sequences selected for human HDAC10 were #2, CAGGTGAACAGTGGTATAGCA, and #3, CGGG TTCTGTGTGTTCAACAA. The sequence targeting the human HDAC10 3′-UTR region was CACCGC AGAAATGACACCGCA. DNA oligonucleotides for these shRNAs were synthesized, annealed, and cloned into pLKO.1, according to the protocol specified by Addgene (Addgene.com). The coding sequence of HDAC10 was amplified by PCR (E003-01A, novoprotein) and cloned into pEGFPC2. EGFP-fusion HDAC10 was subcloned into the FUGW vector with recombinase (NR001A, Novoprotein). The lentiviral plasmid and helper plasmids VSV-G and Pax2 were co-transfected into HEK293T cells with GeneExpresso transfection reagent (EG-1086, Exelgen, USA) to produce lentivirus. The lung cancer cells were infected with the virus in the presence of 8 μl/mg Polybrene.

### Cell proliferation, cell cycle analysis, and cell apoptosis assays

Cell proliferation was evaluated using the Cell Counting Kit-8 assay (CCK8, DOJINDO, Japan). Cells were harvested at 72 hours after virus infection and subjected to propidium iodide staining for cell cycle analysis. Cells were harvested at 96 hours after virus infection and subjected to apoptosis analysis via the Annexin V-FITC Apoptosis Detection Kit (KeyGEN bioTECH). These samples were subjected to FACS analysis.

### Western blotting, immunoprecipitation, immunofluorescence staining

Western blotting was performed as described [36]. The following primary antibodies were used: HDAC10 (H3413, Sigma), P27 (N-20) (sc-527, Santa Cruz), P21 (ab7960, Abcam), Cyclin D1 (sc-718, Santa Cruz), Cyclin E1 (sc-481, Santa Cruz), AKT (#4685S, CST), p-AKT (#4060S, CST), BCL-2 (21060, SAB), BAK (BS1029, Bioworld), Anti-HA (AP0005, Bioworld), GAPDH (sc-47724, Santa Cruz), and Anti-Acetyl-lysine (Millipore, 06-933). The following secondary antibodies were used: HRP-Ms (#7074, CST) and HRP-Rb (#7076, CST). For immunoprecipitation (IP), Anti-HA beads (E6779, Sigma), Anti-M2 beads (F2426, Sigma), normal IgG (#2729, CST), and protein A/G beads (16-125, Millipore) were used. IP was performed as described in [36]. Briefly, the cells were harvested and lysed in lysis buffer (50 mM Tris, pH 7.4, 120 mM NaCl, 0.5 mM EDTA, 0.5% Nonidet P-40, and 15% glycerol) supplemented with protease inhibitors (1 mM phenylmethylsulfonyl fluoride, 0.7 μg/ml pepstatin A, 2 μg/ml aprotinin, 2 mM NaF, and 1 mM Na3VO4). Endogenous AKT or HDAC10 was IPed with antibodies and protein A/G beads. For immunofluorescence staining, the cells were seeded on glass coverslips (12-545-82, Thermo Fisher) and stained as described in [36].

### Tumour xenografts in nude mice

The Institutional Animal Care and Use Committee of Tongji University approved our animal experiments. Five-week-old male nude mice were purchased from the Shanghai Silaike Co. Five mice were injected subcutaneously with 5.0 × 10^6^ cells (for HDAC10 overexpression and its vector control) or 1 × 10^7^ cells (for HDAC10 knockdown and its vector control). The tumours were monitored, and their volumes were calculated at 5-day intervals.
